# Association between serum copper-zinc ratio and respiratory tract infection in children and adolescents

**DOI:** 10.1371/journal.pone.0293836

**Published:** 2023-11-02

**Authors:** Mei Yang, Yanshan Li, Chunlan Yao, Yanzu Wang, Caijin Yan

**Affiliations:** Department of Pediatrics, Xinglin Branch of the First Affiliated Hospital of Xiamen University, Xiamen, Fujian, China; Xi’an Jiaotong University, CHINA

## Abstract

**Background:**

The aim of this study was to explore the association between serum copper-zinc (Cu-Zn) ratio and the risk of respiratory tract infection in children and adolescents.

**Methods:**

This cross-sectional study collected the data of 1695 participants who aged 6–17 years with follow-up data on respiratory tract infection in 2011–2012, 2013–2014 and 2015–2016 cycles from the National Health and Nutrition Examination Survey (NHANES) database. Univariate logistic regression analysis was applied to explore the covariates. Each covariate was adjusted in multivariate logistic regression analysis to explore the correlation between serum Cu-Zn ratio and respiratory tract infection. Subgroup analysis was performed to stratify the data according to age, gender and BMI. Restricted cubic spline (RCS) curve was plotted to identify the association between serum Cu-Zn ratio and respiratory tract infection.

**Results:**

The results of RCS curve depicted that the risk of respiratory tract infection was increased as the elevation of the serum Cu-Zn ratio. After adjusting for confounders, risk of respiratory tract infection in children and adolescents was elevated with the increase of serum copper-zinc ratio (OR = 1.38, 95%CI: 1.19–1.60). Compared with people with serum copper-zinc ratio <1.25, subjects who had serum copper-zinc ratio >1.52 was associated with increased risk of respiratory tract infection in children and adolescents (OR = 1.88, 95%CI: 1.19–2.98). Subgroup analysis demonstrated that the risk of respiratory tract infection was elevated as the increase of serum copper-zinc ratio in participants <12 years (OR = 1.65, 95%CI: 1.28–2.12), ≥12 years (OR = 1.27, 95%CI: 1.03–1.57), males (OR = 1.63, 95%CI: 1.29–2.06), females (OR = 1.26, 95%CI: 1.01–1.57), underweight and normal (OR = 1.35, 95%CI: 1.11–1.65), and overweight and obese participants (OR = 1.44, 95%CI: 1.15–1.80).

**Conclusion:**

Higher serum Cu-Zn ratio was associated with increased risk of respiratory tract infection in children and adolescents, which suggests the importance of Zn supplement and the balance of serum Cu-Zn ratio in children and adolescents.

## Introduction

Respiratory tract infection is a widespread disease among children, and children are the high-risk population for the disease [1]. Respiratory tract infection is an leading cause of hospitalization and mortality in children worldwide [2]. Acute respiratory tract infection was reported to have the highest morbidity and mortality among children younger than 5 years old, with 120–156 million illnesses, and about 920,000 death per year [3, 4]. There was evidence showing that the incidence of respiratory tract infection in children was between 16.8% and 18.7% in China [5]. To explore biomarkers associated with respiratory tract infection to early identify high-risk children is of great value for the management of this disease.

Previously, multiply studies revealed that the immune function, oxidative stress and inflammation status of the body were important factors affecting respiratory tract infection [6, 7]. Minerals are closely related to the immune function, oxidative stress and inflammation of the body [8, 9]. Zinc (Zn) is an important nutrient, and serum zinc level is closely related to immune function [10]. Low serum Zn level is related to pneumonia in infants [11]. Zn supplementation may be protective against respiratory tract infections in Asian children [12]. Additionally, high levels of copper (Cu) have been found to increase the risk of infections such as pneumonia [13]. It is noteworthy that serum Zn and Cu may interact with each other [14]. To consider the effects of zinc and copper in some diseases is of great significance. In recent years, the serum Cu-Zn ratio was identified to more comprehensively reflect the immune level and oxidative stress inflammation state, and was reported to be associated with inflammation and infection-related diseases in adults [15, 16]. The serum Cu-Zn ratio was found to be a an effective biomarker to identify individuals at high risk for severe infections such as pneumonia [13]. Escobedo-Monge et al. depicted that serum Cu-Zn ratio was significantly higher in malaria-infected children [17]. Whether serum Cu-Zn ratio was associated with the risk of respiratory tract infection in children and adolescents was still unclear.

The aim of this study was to explore the association between serum Cu-Zn ratio and the risk of respiratory tract infection in children and adolescents based on the data from the National Health and Nutrition Examination Survey (NHANES) database. We also conducted subgroup analysis in different age, gender and body mass index (BMI) groups.

## Methods

### Study design and population

This cross-sectional study collected the data of 6107 participants who aged 6–17 years with follow-up data on respiratory tract infection in 2011–2012, 2013–2014 and 2015–2016 cycles from the NHANES database. The NHANES database was a nationally representative survey of the civilian, noninstitutionalized US population, which was conducted by the National Center for Health Statistics (NCHS) of the Centers for Disease Control and Prevention (CDC), including demographics, dietary, examination, laboratory, and questionnaire data [18]. In our study, participants without data on Zn and/or Cu, and those without data on BMI were excluded. Finally, 1695 subjects were included. The requirement of ethical approval for this study was waived by the Institutional Review Board of Xinglin Branch of the First Affiliated Hospital of Xiamen University, because the data was accessed from NHANES (a publicly available database). The need for written informed consent from the participants’ legal guardian/next of kin was waived by the Institutional Review Board of Xinglin Branch of the First Affiliated Hospital of Xiamen University due to retrospective nature of the study.

### Potential covariates and definitions

Age (years), gender, race (Mexican American, other Hispanic, non-Hispanic White, non-Hispanic Black, and other race-including multi-racial), family poverty income ratio (PIR), asthma (yes or no), birth weight (<5.5 pounds, 5.5–8.9 pounds, ≥9 pounds or unknown), mother smoked when pregnant (yes, no or unknown), physical activity (yes or no), tobacco smoke exposure (yes or no), energy (kcal), protein (gm), BMI (underweight, normal, overweight or obese), white blood cell (WBC, 1000cells/μL), lymphocyte (%), neutrophils (%), hemoglobin (g/dL), and monocyte (1000cells/uL) were potential covariates.

Asthma was defined based on the answer of “yes” to variable MCQ010 (Ever been told you have asthma). Birth weight was identified according to the variable ECD070A, and was divided into <5.5 pounds, 5.5–8.9 pounds, ≥9 pounds. Mother smoked when pregnant was found based on the answer of “yes” to variable ECQ020. Tobacco smoke exposure was defined according to the variable LBXCOT of cotinine in the database, LBXCOT > 0.05μg/L was regarded to have tobacco smoke exposure. Physical activity was defined according to the variable PAQ706, and the record of 1–7 was considered to have physical activity in children and adolescents < 12 years; In children and adolescents of 12–17 years, the answer of “yes” to variable PAQ605 or PAQ650 was considered to have physical activity. Obesity was defined as a BMI at or above the 95th percentile of the CDC sex-specific BMI-for-age growth charts from 2000 [19]. Overweight was defined as a BMI between the 85th and 95th percentiles, normal was defined as a BMI between the 5^th^, and 85th percentiles and those with a BMI <5th percentiles was divided into the underweight group.

### Main and outcome variables

The serum Cu-Zn ratio was the main variable in the present study. The serum Cu level was identified based on the variable LBXSCU and the serum Zn level was identified based on the variable LBXSZN in the NHANES database. The serum Cu-Zn ratio was divided into <1.25 group, 1.25–1.52 group, and >1.52 group according to their tertiles.

Whether participants had respiratory tract infection was the outcome. Participants answered “yes” to any of the variable HSQ500 (have a head cold or chest cold that started during those 30 days) or HSQ520 (have flu, pneumonia, or ear infections that started during those 30 days; or ear infections that started during those 30 days) were considered to have a respiratory tract infection.

### Statistical analysis

All estimates were calculated accounting for NHANES sample weights (sdmvstra and sdmvpsu). The masked variance unit pseudo-stratum was sdmvstra, and the masked variance unit pseudo-primary sampling units was sdmvpsu with the confidence interval (CI) was applied for evaluating the reliability of an estimate. The measurement data were described as weighted Mean and standard error (S.E), and the comparison between groups was performed by variance analysis. The enumeration data were described as unweighted numbers (weighted percentages) [n (%)]. Chi-square test or Fisher’s exact probability method were used for comparison between groups. Missing values were manipulated (Table 1). The weighted univariate weighted logistic regression analysis was applied to explore the covariates. Each covariate was adjusted in the weighted multivariate logistic regression analysis to explore the correlation between serum Cu-Zn ratio and respiratory tract infection. Subgroup analysis was performed to stratify the data according to age, gender and BMI. Restricted cubic spline (RCS) curve was plotted to identify the association between serum Cu-Zn ratio and respiratory tract infection. The odds ratio (OR) and 95% CI were used as the effect size. Confidence level was set as alpha = 0.05. SAS9.4 software (SAS Institute Inc., Cary, NC, USA) was used for data extraction, and statistical analysis. RCS curve was drawn using R version 4.2.2 (2022-10-31 ucrt).

**Table 1:** The number and percentages of missing values.

Variables	n	Percentage (%)
Ratio of family income to poverty	123	7.26
Tobacco smoke exposure	29	1.71
Physical activity	43	2.54
Energy	36	2.12
Protein	36	2.12
WBC	7	0.41
Lymphocyte	9	0.53
Neutrophils	9	0.53
Hemoglobin	7	0.41
Monocyte	9	0.53

WBC: White blood cells count

## Results

### Comparisons of the characteristics of participants in different serum Cu-Zn ratio groups

**Fig 1:**
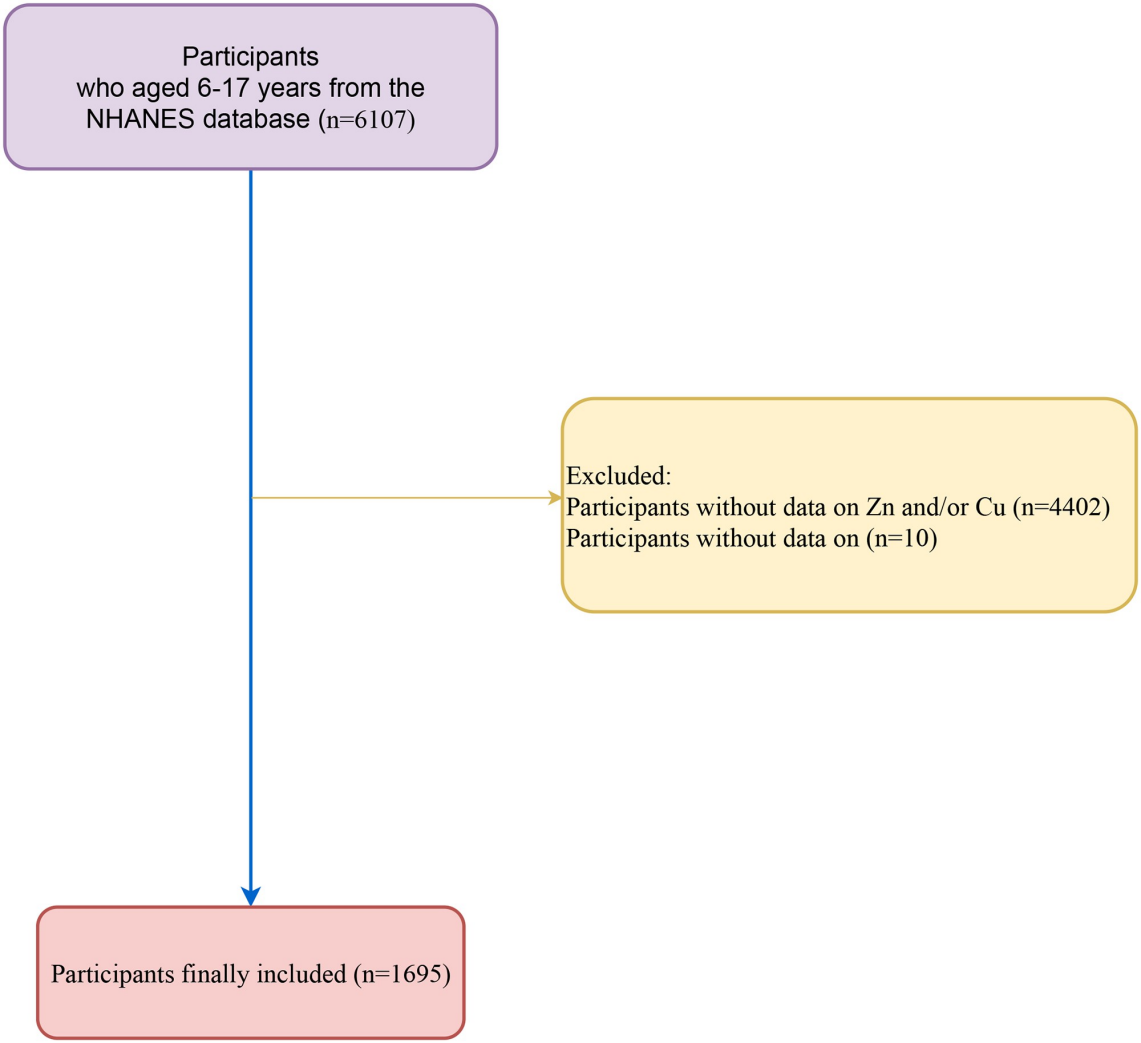
The screen process of the participants.

In total, the data of 6107 participants who aged 6–17 years with follow-up data on respiratory tract infection from the NHANES database. Among them participants without data on Zn and/or Cu and those without data on BMI were excluded. Finally, 1695 subjects were included. The screen process was exhibited in Fig 1.

The percentages of participants < 12 years were statistically different among Cu-Zn ratio <1.25, 1.25–1.52, and >1.52 groups (25.93% vs 46.33% vs 58.51%). The energy in the Cu-Zn ratio <1.25 group was higher than Cu-Zn ratio 1.25–1.52, and >1.52 groups (21.50.90 kcal vs 1995.80 kcal vs 1936.59 kcal). The percentages of subjects in different race, and BMI groups were statistically different among Cu-Zn ratio <1.25, 1.25–1.52, and >1.52 groups. The percentage of participants with respiratory tract infection in the Cu-Zn ratio >1.52 group was higher than Cu-Zn ratio <1.25, and 1.25–1.52 group (30.09% vs 16.19% vs 15.22%) (Table 2).

**Table 2:** The characteristics of participants.

Variables	Total n = 1695	Serum Cu-Zn ratio			Statistics	*P*
		<1.25 n = 546	1.25–1.52 n = 528	>1.52 n = 621		
Age, n (%)					χ^2^ = 72.406	<0.001
<12 years	849 (43.61)	172 (25.93)	272 (46.33)	405 (58.51)		
≥12 years	846 (56.39)	374 (74.07)	256 (53.67)	216 (41.49)		
Gender, n (%)					χ^2^ = 11.772	0.003
Male	842 (50.70)	299 (58.20)	243 (44.03)	300 (49.91)		
Female	853 (49.30)	247 (41.80)	285 (55.97)	321 (50.09)		
Race/Hispanic origin, n (%)					χ^2^ = 22.649	0.004
Mexican American	387 (16.21)	127 (17.26)	130 (15.30)	130 (16.07)		
Other Hispanic	194 (7.72)	61 (7.69)	58 (7.15)	75 (8.33)		
Non-Hispanic White	429 (52.73)	142 (51.41)	141 (57.16)	146 (49.63)		
Non-Hispanic Black	419 (13.69)	104 (10.92)	119 (11.99)	196 (18.13)		
Other Race—Including Multi-Racial	266 (9.65)	112 (12.72)	80 (8.40)	74 (7.85)		
Ratio of family income to poverty, Mean (S.E)	2.40 (0.08)	2.33 (0.11)	2.57 (0.11)	2.31 (0.13)	F = 2.27	0.114
Asthma, n (%)					χ^2^ = 3.496	0.174
No	1360 (79.50)	451 (83.01)	421 (79.00)	488 (76.50)		
Yes	335 (20.50)	95 (16.99)	107 (21.00)	133 (23.50)		
Birth weight, n (%)					χ^2^ = 23.333	<0.001
<5.5 pounds	196 (10.78)	50 (8.95)	66 (10.96)	80 (12.43)		
5.5–8.9 pounds	1041 (60.46)	297 (53.55)	335 (65.33)	409 (62.47)		
≥9 pounds	132 (7.89)	44 (7.33)	42 (7.87)	46 (8.47)		
Unknown	326 (20.87)	155 (30.17)	85 (15.84)	86 (16.63)		
Mother smoked when pregnant, n (%)					χ^2^ = 18.736	<0.001
No	1236 (70.41)	359 (63.45)	398 (74.02)	479 (73.71)		
Yes	160 (10.64)	41 (8.86)	52 (11.58)	67 (11.46)		
Unknown	299 (18.96)	146 (27.69)	78 (14.39)	75 (14.83)		
Physical activity, n (%)					χ^2^ = 3.535	0.171
No	202 (11.79)	83 (14.52)	62 (10.64)	57 (10.22)		
Yes	1493 (88.21)	463 (85.48)	466 (89.36)	564 (89.78)		
Tobacco smoke exposure, n (%)					χ^2^ = 1.058	0.589
No	1014 (62.00)	341 (63.33)	328 (62.74)	345 (59.95)		
Yes	681 (38.00)	205 (36.67)	200 (37.26)	276 (40.05)		
Energy, kcal, Mean (S.E)	2027.59 (25.45)	2150.90 (55.03)	1995.80 (45.32)	1936.59 (39.36)	F = 4.09	0.023
Protein, gm, Mean (S.E)	70.57 (1.08)	77.61 (2.37)	67.70 (1.52)	66.43 (1.54)	F = 7.57	0.001
BMI, n(%)					χ^2^ = 43.404	<0.001
Underweight	42 (2.73)	17 (2.79)	15 (4.02)	10 (1.38)		
Healthy Weight	942 (56.47)	372 (66.83)	294 (53.49)	276 (49.13)		
Overweight	298 (18.41)	82 (15.97)	98 (20.44)	118 (18.80)		
Obese	413 (22.39)	75 (14.41)	121 (22.05)	217 (30.69)		
WBC, 1000cells/μl, Mean (S.E)	7.01 (0.08)	6.30 (0.10)	7.03 (0.10)	7.72 (0.12)	F = 57.62	<0.001
Lymphocyte, %, Mean (S.E)	36.68 (0.31)	36.55 (0.48)	37.57 (0.69)	35.92 (0.39)	F = 2.48	0.095
Neutrophils, %, Mean (S.E)	51.04 (0.33)	51.11 (0.48)	50.17 (0.74)	51.84 (0.48)	F = 2.19	0.123
Hemoglobin, g/dL, Mean (S.E)	13.56 (0.05)	14.06 (0.08)	13.57 (0.08)	13.03 (0.06)	F = 92.73	<0.001
Monocyte, 1000cells/uL, Mean (S.E)	0.56 (0.01)	0.51 (0.01)	0.55 (0.01)	0.62 (0.01)	F = 22.32	<0.001
Respiratory tract infection, n (%)					χ^2^ = 24.905	<0.001
No	1362 (79.49)	468 (83.81)	438 (84.78)	456 (69.91)		
Yes	333 (20.51)	78 (16.19)	90 (15.22)	165 (30.09)		

S.E: standard error, BMI: Body Mass Index; WBC: White blood cells count

### Association between serum copper-zinc ratio and respiratory tract infection in children and adolescents

**Fig 2:**
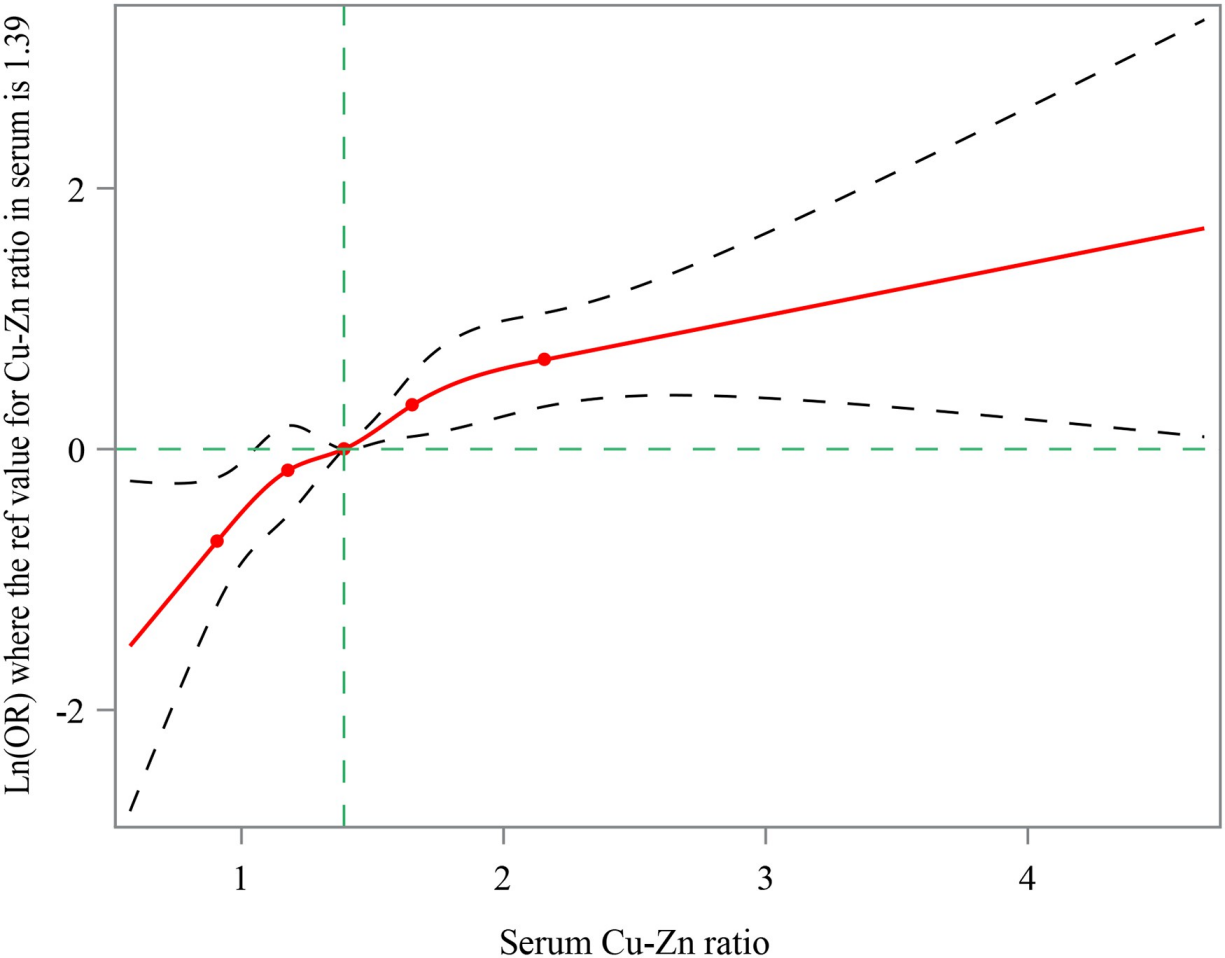
RCS curve depicting the risk of respiratory tract infection was increased as the elevation of the serum Cu-Zn ratio.

The results of RCS curve depicted that the risk of respiratory tract infection was increased as the elevation of the serum Cu-Zn ratio (Fig 2). As exhibited in Table 3, asthma (OR = 1.64, 95%CI: 1.04–2.59), tobacco smoke exposure (OR = 1.41, 95%CI: 1.02–1.93), normal weight (OR = 1.62, 95%CI: 1.08–2.44), WBC (OR = 1.28, 95%CI: 1.11–1.48), and monocyte (OR = 1.25, 95%CI: 1.10–1.41) were confounding factors associated with the risk of respiratory tract infection in children and adolescents. After adjusting for these confounders, the risk of respiratory tract infection in children and adolescents was elevated with the increase of serum copper-zinc ratio (OR = 1.38, 95%CI: 1.19–1.60). Compared with people with serum copper-zinc ratio <1.25, subjects who had serum copper-zinc ratio >1.52 was associated with increased risk of respiratory tract infection in children and adolescents (OR = 1.88, 95%CI: 1.19–2.98). Sensitivity analysis revealed that the results showed no significant difference before and after missing values inputting (Table 4).

**Table 3:** Association between serum copper-zinc ratio and respiratory tract infection in children and adolescents.

Variables	Crude model		Multivariate Model	
	OR (95%CI)	*P*	OR (95%CI)	*P*
Serum Cu-Zn ratio	1.44 (1.24–1.68)	<0.001	1.38 (1.19–1.60)	<0.001
Serum Cu-Zn ratio				
<1.25	Ref		Ref	
1.25–1.52	0.93 (0.56–1.53)	0.769	0.86 (0.52–1.41)	0.535
>1.52	2.23 (1.42–3.49)	<0.001	1.88 (1.19–2.98)	0.008

Ref: Reference, OR: Odds Ratio, CI: Confidence Interval

Multivariate model adjusted for asthma, tobacco smoke exposure, BMI, WBC, and monocyte.

**Table 4:** Sensitivity analysis of the results before missing value manipulation.

Variables	Multivariable model before manipulation		Multivariable Model after manipulation	
	OR (95%CI)	*P*	OR (95%CI)	*P*
Serum Cu/Zn ratio	1.38 (1.19–1.60)	<0.001	1.38 (1.19–1.60)	<0.001
Serum Cu/Zn ratio				
<1.25	Ref		Ref	
1.25~1.52	0.88 (0.53–1.45)	0.606	0.86 (0.52–1.41)	0.535
>1.52	1.90 (1.20–3.00)	0.007	1.88 (1.19–2.98)	0.008

Ref: Reference, OR: Odds Ratio, CI: Confidence Interval

Multivariate model adjusted for asthma, tobacco smoke exposure, BMI, WBC, and monocyte

**Fig 3:**
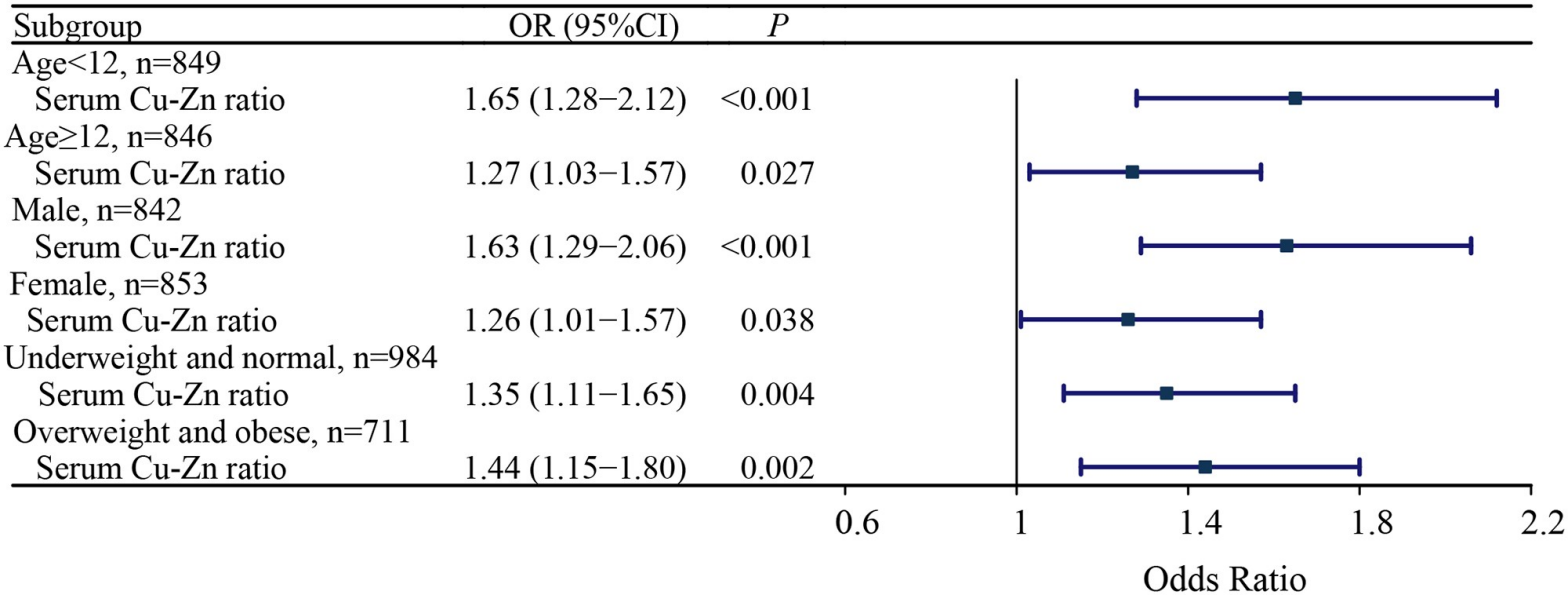
Forest plot showing the association between serum copper-zinc ratio and respiratory tract infection in children and adolescents in different subgroups.

Subgroup analysis demonstrated that the risk of respiratory tract infection was elevated as the increase of serum copper-zinc ratio in participants <12 years (OR = 1.65, 95%CI: 1.28–2.12), ≥12 years (OR = 1.27, 95%CI: 1.03–1.57), males (OR = 1.63, 95%CI: 1.29–2.06), females (OR = 1.26, 95%CI: 1.01–1.57), underweight and normal (OR = 1.35, 95%CI: 1.11–1.65), and overweight and obese (OR = 1.44, 95%CI: 1.15–1.80) (Fig 3).

## Discussion

The current study analyzed the association between serum Cu-Zn ratio and the risk of respiratory tract infection in children and adolescents based on the data from the NHANES database. The results demonstrated that higher serum Cu-Zn ratio was associated with increased risk of respiratory tract infection in children and adolescents. Those with serum copper-zinc ratio >1.52 was associated with increased risk of respiratory tract infection in children and adolescents. Subgroup analysis concerning different ages, genders and BMI also identified similar results. The findings suggested the potential role of evaluating Cu-Zn ratio for the prevention of respiratory tract infection in children and adolescents and also the importance of nutrition balance of children and adolescents.

Cu and Zn are essential micronutrients involved in several cellular processes and different diseases [20, 21]. Previously, Malavolta et al. depicted that the increase of Cu-Zn ratio was identified in several age-related chronic diseases [22]. A recent prospective study indicated that serum Cu-Zn ratio was correlated with the risk of incident infections including acute respiratory infections, other bacterial diseases, viral diseases, influenza, and pneumonia in men [15]. However, the outcome was not specified, whether the association between serum Cu-Zn ratio and the risk of acute respiratory infections could be obtained still needed exploration [15]. Another prospective cohort study indicated that serum Cu-Zn ratio was associated with the risk of incident pneumonia in Caucasian men, which was a better risk indicator than high-sensitivity C-reactive protein (hs-CRP) [13]. The Cu/Zn ratio was relatively high in infected newborns and was positively correlated with C-reactive protein (CRP) levels, and the OR for the Cu-Zn ratio was 9.067, indicating a higher probability of infection [23]. In the present study, higher serum Cu-Zn ratio was associated with increased risk of respiratory tract infection in children and adolescents.

The potential mechanisms underlying our findings might be that acute infections might due to the increase of the serum and local concentration of Cu, leading to Cu toxicity in the pathogen [24] and the decrease of serum Zn due to redistribution through the activation of inflammatory cytokines in some tissues [25]. These two responses to inflammation may impair the immune defense and increase the risk of infection, especially in vulnerable patients, such as children with an immature immune system [26]. Copper was identified as a pro-oxidant and in the metal accelerated production of free radicals affecting oxidative stress, while Zn was an antioxidant, and its changes may affect the homeostasis of oxidative stress [27]. Acute infections alter micronutrients metabolism, and the deficiencies of micronutrients also increased the risk of infection, which indicated Cu-Zn ratio might be a significant diagnostic biomarker for early onset infections [26]. Nutrition is not only essential to reduce the risk of an individual’s susceptibility to infection, but also improves the prevention and treatment of diseases, as well as modifies the course of diseases, especially among children [28]. The findings of our study might provide a reference for improving the awareness of supplying of Zn in children and adolescents, and keeping the balance of Cu and Zn in children and adolescents.

Our study analyzed the data from the NHANES database, and multi-stage complex sampling was adopted with good sample representativeness. There were several limitations. Firstly, this study was a cross-sectional study and could not suggest a causal association between Cu-Zn ratio and the risk of respiratory tract infection in children and adolescents. Secondly, the type and severity of respiratory tract infection were not collected in the database, and the relationship of serum Cu-Zn ratio with different types and severity of respiratory tract infection needs further exploration.

## Conclusions

This study evaluated the association between serum Cu-Zn ratio and the risk of respiratory tract infection in children and adolescents. The findings showed that higher serum Cu-Zn ratio was associated with increased risk of respiratory tract infection in children and adolescents. The results might suggest the importance of Zn supplement in children and adolescents, and keep the balance of serum Cu-Zn ratio is also important.

## Supporting information

S1 Checklist STROBE statement—Checklist of items that should be included in reports of observational studies. (DOCX)
